# Expression of Dominant-Negative Thyroid Hormone Receptor Alpha1 in Leydig and Sertoli Cells Demonstrates No Additional Defect Compared with Expression in Sertoli Cells Only

**DOI:** 10.1371/journal.pone.0119392

**Published:** 2015-03-20

**Authors:** Betty Fumel, Pascal Froment, Martin Holzenberger, Gabriel Livera, Philippe Monget, Sophie Fouchécourt

**Affiliations:** 1 INRA, UMR85 Physiologie de la Reproduction et des Comportements, F-37380, Nouzilly, France; 2 CNRS, UMR7247 Physiologie de la Reproduction et des Comportements, F-37380, Nouzilly, France; 3 Université François Rabelais de Tours, F-37041, Tours, France; 4 IFCE, F-37380, Nouzilly, France; 5 INSERM and Sorbonne Universités—UPMC, UMRS 938, Hôpital Saint-Antoine, 75012, Paris, France; 6 Laboratoire de Développement des Gonades, INSERM U967, CEA/DSV/iRCM/SCSR/LDG, Univ Paris Diderot, Sorbonne Paris Cité, Univ Paris Sud, F-92265, Fontenay-Aux-Roses, France; Clermont Université, FRANCE

## Abstract

**Background:**

In the testis, thyroid hormone (T3) regulates the number of gametes produced through its action on Sertoli cell proliferation. However, the role of T3 in the regulation of steroidogenesis is still controversial.

**Methods:**

The TRα^AMI^ knock-in allele allows the generation of transgenic mice expressing a dominant-negative TRα1 (thyroid receptor α1) isoform restricted to specific target cells after Cre-loxP recombination. Here, we introduced this mutant allele in both Sertoli and Leydig cells using a novel aromatase-iCre (ARO-iCre) line that expresses Cre recombinase under control of the human Cyp19(IIa)/aromatase promoter.

**Findings:**

We showed that loxP recombination induced by this ARO-iCre is restricted to male and female gonads, and is effective in Sertoli and Leydig cells, but not in germ cells. We compared this model with the previous introduction of TRα^AMI^ specifically in Sertoli cells in order to investigate T3 regulation of steroidogenesis. We demonstrated that TRα^AMI^-ARO males exhibited increased testis weight, increased sperm reserve in adulthood correlated to an increased proliferative index at P3 *in vivo*, and a loss of T3-response *in vitro*. Nevertheless, TRα^AMI^-ARO males showed normal fertility. This phenotype is similar to TRα^AMI^-SC males. Importantly, plasma testosterone and luteinizing hormone levels, as well as mRNA levels of steroidogenesis enzymes StAR, Cyp11a1 and Cyp17a1 were not affected in TRα^AMI^-ARO.

**Conclusions/Significance:**

We concluded that the presence of a mutant TRα^AMI^ allele in both Leydig and Sertoli cells does not accentuate the phenotype in comparison with its presence in Sertoli cells only. This suggests that direct T3 regulation of steroidogenesis through TRα1 is moderate in Leydig cells, and that Sertoli cells are the main target of T3 action in the testis.

## Introduction

Thyroid hormones, mainly represented by triiodothyronine (T3), and their nuclear receptors play important roles in the development, differentiation and function of various organs, including the male reproductive system [1,2]. The *THRA/NR1A1* and *THRB/NR1A2* genes encode the T3 nuclear receptors (TRs): TRα1 (thyroid hormone receptor α1) for *THRA*, and TRβ1 and TRβ2 for *THRB*. TRα1 and TRβ1 are expressed ubiquitously. In the testes, T3 is of particular importance as it regulates the number of gametes produced and the size of the gonad [3]. It has been proposed that T3 regulates both Sertoli cells (SC), which are specialized supporting cells for germ cells, and Leydig cells (LC) that produce testosterone. The T3 effect related to SC is well-known. Indeed, pharmacological approaches [4–6] led to the conclusion that T3 controls post-natal SC proliferation *in vivo*. Thereafter, the functional study with homozygous mice bearing a null deletion of the *THRA* gene (TRα^Null/Null^ mice) showed that TRα1 mediates this control [7]. More recently, we demonstrated, using mice with SC-specific TRα1 receptor dominant-negative expression (TRα^AMI^-SC mice) [8,9], that T3 exerts this regulation in a direct cell-autonomous manner. Adult LC are the primary source of androgens in mature mammalian testes, but controversy exists regarding T3-regulation of steroidogenesis. LC arise from pluripotent mesenchymal precursors (for review see [10,11]), and start to differentiate at post-natal day 14 (P14) in rats [12] and P10 in mice [13], and gradually mature to adult LC just before puberty. The effect of T3 on LC has been mostly investigated in rat species, using *in vitro* and *in vivo* pharmacological studies. It is established that T3 is important for mesenchymal stem cell proliferation and differentiation into LC. Indeed, *in vivo*, exogenous T3 in neonate rats leads to an increase in differentiated LC numbers and influences their proliferation [14]. The overall impact on steroidogenesis at adulthood is still a matter of debate. *In vitro* studies using LC cultures suggest that T3 directly increases steroidogenesis [15]. In rats with pharmacologically induced hypothyroidism during post-natal development, it has been observed that peripheral testosterone concentrations were unchanged [4,16,17]. However, studies on neonatal hypothyroid rats showed a decrease in blood testosterone levels at adulthood [18,19]. Thus, there is a debate about possible T3 regulation of steroidogenesis. Unfortunately, no genetic evidence supports this matter. In the two previously cited transgenic mouse models (TRα^Null/Null^ and TRα^AMI^-SC), we showed that testosterone levels were unchanged. Nevertheless, as the modification was restricted to SC in TRα^AMI^-SC mice and ubiquitously present in TRα^Null/Null^ mice, these two transgenic lines were not well suited to investigate direct T3 regulation of steroidogenesis by this receptor.

The ubiquitous TRα1 receptor is present in rat LC [20], but no data are yet available in mice. Following our functional study in TRα^AMI^-SC mice [8], we aimed to investigate the role of the TRα1 receptor in the regulation of steroidogenic activity using a novel functional Cre transgenic mouse, the aromatase-iCre (ARO-iCre), that we generated in our laboratory and characterized for this study. After demonstrating the pertinence of this line for Cre-loxP recombination in both SC and LC, we produced TRα^AMI^-ARO mice expressing dominant negative TRα^AMI^ selectively in SC and LC and compared their phenotypes with the previously established TRα^AMI^-SC line at cellular and endocrine levels. The present study confirms our findings, namely that T3 influences Sertoli cell proliferation through its TRα1 receptor. Moreover, we showed that the expression of dominant-negative TRα^AMI^ in LC does not influence testosterone production in adulthood.

## Materials and Methods

### Animals

Mice were maintained under standard conditions of light (12 h light, 12 h darkness) and temperature (21–23°C) with *ad libitum* access to food and water. All animal studies were conducted in accordance with the guidelines for the care and use of laboratory animals issued by the French Ministry of Agriculture and with the approval of a local ethical review committee, under project number 2011-09-10 (Comité d’Ethique en Expérimentation Animale Val de Loire- n°19). The TRα^AMI^-ARO line was produced and investigations were performed at the same time as the TRα^AMI^-SC, except for steroidogenic enzyme determination. Mice were euthanized by cervical dislocation under anesthesia with ketamine (87 μg/g body weight) and xylazine (13 μg/g body weight) by intraperitoneal injection. All efforts were made to minimize animal stress and suffering.

### Production of aromatase-iCre (ARO-iCre) line

The aromatase-iCre transgene was generated by placing the cDNA encoding a mammalian codon-improved Cre recombinase (iCre: [21,22]) followed by an SV40 poly(A) signal under control of the human Cyp19/aromatase(IIa)_-278_ promoter cloned by Hinshelwood et al. This promoter is sufficient to mediate restrictive transgenic expression in the ovary and the testis [23]. This region contains two response elements, a steroidogenic factor 1-(SF1)-site and a CRE-like sequence binding CREB protein, critical for cAMP induction of human Cyp19/aromatase promoter activity.

**Fig 1:**
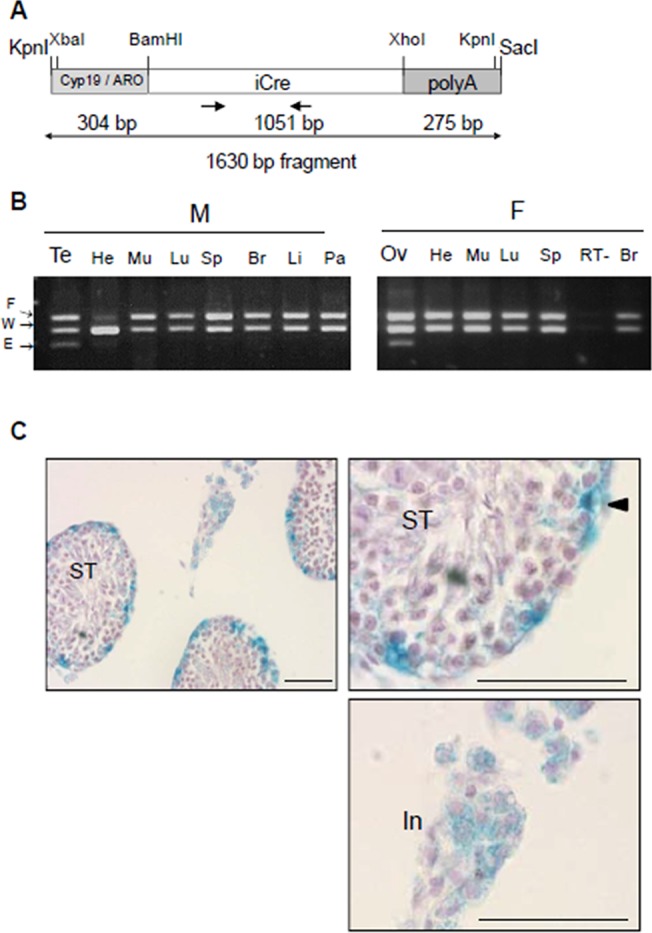
Production and characterization of ARO-iCre transgenic mouse line. **(a)** Schematic diagram of the DNA construct used for producing ARO-iCre mice. The mammalian codon-improved Cre recombinase (iCre, 1051 bp; white box) followed by an SV40 poly(A) signal cassette (right grey box) is controlled by 304 bp of the human Cyp19/aromatase(IIa)_-278_ promoter (left grey box). Arrows indicate position and direction of primers. Oligonucleotide sequences are in Table 1. **(b)** Cre excision is restricted to ovaries (Ov) and testes (Te) of iCre^+/0^;IGF1R^flox/WT^ mice. The excision was only observed in gonads, not in other tissues (He, heart; Mu, muscle; Lu, lung; Sp, spleen; Br, brain; Li, liver; Pa, pancreas) from males (M) and females (F). RT-, negative control (testis cDNA without RT). PCR detected wild-type (W, 256 bp) and floxed (F, 312 bp) alleles in all tissues, and the excised allele (E, 204 bp) exclusively in gonads. **(c)** Cre recombinase activity detected in somatic cells of testes in adult ARO-iCre males. After crossing the ARO-iCre mice with a ROSA26 Cre reporter mouse, β-galactosidase activity was detected in both SC and LC. No activity was present in germ cells. Right micrograph, low magnification; left micrographs, details in higher magnification. ST, seminiferous tubules. In, interstitium. Arrow head points to SC. Bar represents 50 μm.

To obtain a Cyp19/aromatase-iCre plasmid, the pUChGX plasmid containing the Cyp19/aromatase(IIa)_-278_ promoter (a 304 bp fragment stretching between position -278 of 5’-flanking DNA and +26 of untranslated exon IIa) was *Hin*dIII digested, blunted and *Bam*HI digested. The fragment was cloned into the blunted *Xba*I and *Bam*HI sites of the pBlue-iCre vector. Next, a fragment containing the SV40 poly(A) signal was obtained by *Hin*dIII digestion, end blunting and *Kpn*I digestion of the pGEM3Zf-polyA (SV40) plasmid and inserted into the blunted *Xho*I and *Kpn*I sites of the Cyp19/aromatase-iCre vector. All DNA constructs were confirmed by sequencing. The 1,630 bp purified fragment containing the Cyp19/aromatase-iCre-polyA(SV40) construction was obtained through *Kpn*I and *Sac*I digestion (Fig. 1A). The transgene was injected into the pronuclei of fertilized eggs from mice with a hybrid DBA/B6 genetic background. Three founder animals (two males and one female) with genomic transgene integration were detected by PCR as described [24]. Specificity of ARO-iCre loxP excision was investigated at tissue level by genomic PCR and at cellular level using Cre-loxP reporter mice. Offspring of all 3 founders targeted Cre specifically to somatic cells of the gonads, without producing excision in germ cells. We chose the strain that transmitted the transgene with highest efficiency to the descendants. This line was called ARO-iCre.

**Table 1:** Primers for PCR genotyping of TRα^AMI^-ARO line and for RT-PCR of TRα1 [27] and actin genes.

Gene	Forward PCR primer (5’-3’)	Reverse PCR primer (5’-3’)	Temp.	Length
TRα^AMI^	GGTTGGTCCAAGGAAAGACA	GCTTCTTGCCGTTTGAAGAC	60°C	520 bp
iCre	CCTGGAAGATGCTCCTGTCTG	AGGGTGTTGTAGGCAATGCC	58°C	391 bp
Actin	TACGACCAGAGGCATACAGG	TGACCCAGATCATGTTTGAGA	55°C	411 bp
TRα1	TGCCTTTAACCTGGATGACAC	TCGACTTTCATGTGGAGGAAG	60°C	720 bp

### Generation of TRα^AMI^-ARO and control mice

The TRα^AMI^ allele generated by Quignodon et al. [25] encodes a TRα1 receptor with a point mutation changing a leucine to an arginine in the AF-2 domain of the receptor (L400R). This mutation prevents the recruitment of histone acetyltransferase coactivators, while interaction with histone deacetylase corepressors is preserved, resulting in dominant-negative activity. The advantage of this mutation is that repression of transcription is maintained even in the presence of T3, with heterozygosity being sufficient to achieve the dominant-negative effect. This mutation is activated by Cre-loxP recombination [25]. According to the procedure we used to establish TRα^AMI^-SC from AMH-Cre and TRα^AMI/AMI^ lines [8], we crossed homozygous TRα^AMI/AMI^ mice (B6/129Sv genetic background) with hemizygous ARO-iCre mice to obtain TRα^AMI/WT^;ARO-iCre^+/0^ mice, hereafter called TRα^AMI^-ARO. These double transgenic mice efficiently express the targeted dominant-negative TRα1 isoform in SC and in LC. TRα^AMI/WT^ littermates that tested negative for ARO-iCre were used as control mice throughout this study. Genotyping of the mice was performed using the primers for iCre and TRα^AMI^ alleles listed in Table 1.

### Fertility, testis weight and sperm reserve

TRα^AMI^-ARO mice and their controls were sacrificed and one testis weighed and frozen for sperm reserve determination as described for TRα^AMI^-SC [8]. Briefly, testes were disrupted in 3 ml of L15 medium (Gibco-Invitrogen) and sonicated for 30 s. Remaining sperm nuclei were counted using a hemocytometer method. These nuclei contain spermatozoa and stage II-VII elongating spermatid nuclei, and their number defines the testicular sperm reserve [3]. Each male (n = 10 per genotype) was mated with two primiparous Swiss female mice. The birth dates were noted to detect a putative delay in mating, and pups were counted at birth.

### Histology

Testes were fixed in Bouin’s solution and embedded in paraffin. Sections 4 μm thick were stained with hematoxylin for microscopic observation of seminiferous tubule organization.

### Determination of SC proliferation index *in vivo* and *in vitro*

Procedures were previously described for TRα^AMI^-SC [8]. Briefly, TRα^AMI^-ARO mice at P3 were injected with 50 μg per gram of body weight of 5-bromo-2-deoxyuridine (BrdU, Sigma) 3 h before sacrifice. After fixation in Bouin’s solution and embedding in paraffin, BrdU immunodetection was performed (monoclonal antibody from Roche; 1:200). A total of 1000 proliferating (BrdU-stained) and non-replicating (BrdU-negative) SC were counted per animal using Histolab analysis software (GT Vision). For organotypic cultures, TRα^AMI^-ARO testes at P3 were cut into small pieces. Culture conditions were as described previously [26], except that T3 (0.2 μM) or a vehicle (1X PBS with 0.025 N NaOH) was added to the medium. Explants were cultured for 72 h and BrdU was added at a final concentration of 0.01 mg/ml after 69 h (i.e. 3 h prior to fixation, exactly as *in vivo*). The explants were then fixed in Bouin’s fixative and processed for BrdU staining.

### RNA extraction, reverse transcription and PCR for TRα1 detection

Total RNA was isolated from whole testes at post-natal day 0 (P0), 3 (P3), 10 (P10), 22 (P22) or adulthood (pool of 3 animals per age) and from Sertoli and Leydig cell-enriched fractions obtained at P10 and prepared as described previously [8]. RNA (1 μg) was reverse transcribed using the RNeasy kit (with DNAse I) according to the manufacturer’s instructions (Qiagen). A sample with RNA but without reverse transcriptase (RT-) served as a negative control. TRα1 mRNA was detected as described [27] by classical PCR using primers and hybridization temperatures as described in Table 1. The β-actin gene was used to control RNA quality.

### Quantitative real-time PCR for analysis of testicular steroidogenesis

Three key steroidogenic genes were analyzed using the TaqMan assay, with primers and probes inventoried by Applied Biosystems: StAR (Steroidogenic acute regulatory protein), Mm00441558_m1; Cyp11a1/P450scc (cytochrome P450 side-chain cleavage enzyme), Mm00490735_m1; Cyp17a1/P450c17 (steroid 17 α hydroxylase/17,20 lyase cytochrome P450c17), Mm00484040_m1. PCR reagents were purchased from Applied Biosystems and real-time PCR carried out in accordance with the manufacturer’s instructions, in a final volume of 25 μl. Samples were analyzed in triplicate. Fluorescence was detected on an iCycler BioRad apparatus. Negative controls (RT- and H_2_0) were included for every primer/probe combination. Normalization was performed using two internal standards, β-actin (Mm00607939_s1) and Gapdh (m99999915_g1) from the same sample. The normalized cDNA was compared between the two genotypes.

### Blood collection and hormone assays

Around 500 μl of blood were obtained by retro-orbital sampling in anesthetized mice (with ketamine 87 μg/g body weight and xylazine 13 μg/g body weight; intraperitoneal injection) and collected in a tube containing EDTA (0.13 M). For plasma testosterone determinations, adult mice were treated with an intraperitoneal injection of 15 IU of human chorionic gonadotropin (hCG) (Chorulon). Blood was collected before (basal level) and 2 h after injection (stimulated level). Plasma was stored at -20°C until tritium-based testosterone competitive radioimmunoassays. RIA are carried out regularly in the lab and were performed as described [8]. The sensitivity of the assay was 0.125 ng/ml. The intra-assay coefficient of variation was 7.5%. Briefly, samples (two different dilutions per sample) or testosterone dilutions (to determine the range) were incubated for 1 h at 40°C (0.1 M phosphate buffer, 0.1% gelatin) with tritiated testosterone plus the anti-testosterone antibody. A secondary antibody was added and the mixtures incubated overnight at 4°C. Subsequent immuno-precipitation was performed with PEG (polyethylene glycol) 4000 and the radioactivity was counted (Packard C2900 TriCarb). Plasma LH (luteinizing hormone) levels were determined in a volume of 50 μl using the immuno-enzymatic LH-DETECTkit for rodents from ReprodPharm, according to manufacturer’s instructions, except that the antibody was used at 4°C overnight, with tetramethylbenzidine as the substrate.

### Statistical analysis

All data are presented as mean ± SEM. To compare means between two groups, Student’s *t*-test or Mann-Whitney *U* test, in case of differences in variance (Fisher’s exact test), was used. Other comparisons were performed using a two-way ANOVA followed by the Bonferroni *post-hoc* test. *P* < 0.05 was considered significant.

## Results

### Generation of an ARO-iCre transgenic strain

ARO-iCre transgenic mice were generated as indicated in materials and methods (Fig. 1A). We characterized the ARO-iCre line first by crossing iCre positive animals with mice carrying a floxed conditional gene. Monitoring Cre excision with the TRα^AMI^ allele is technically challenging because it only diverges from the wild-type TRα1 by a single amino acid mutation [25]. Therefore, we used the floxed allele of the insulin-like growth factor-1 receptor (IGF1R) gene [28–30] for characterization. IGF1R excision was analyzed in various organs of iCre^+/0^;IGF1R^flox/WT^ pups using triplex PCR on genomic DNA [24]. We found the excised allele exclusively in samples from male and female gonads (Fig. 1B), confirming the promoter characterization of Hinshelwood et al. [23] and demonstrating the usefulness of the transgenic line for functional gonad investigation. In particular, we did not observe any excision in the brain (Fig. 1B), nor in the pituitary (not shown). To characterize the ARO-iCre transgenic line at cellular level, we crossed it to mice containing the ROSA26 Cre reporter (*R26R*) allele [31]. β-galactosidase (β-gal) activity resulting from loxP recombination at the *R26R* locus by ARO-iCre recombinase was observed at the periphery of the seminiferous tubules in SC and in the interstitium containing LC (Fig. 1C). In contrast, no activity was present in germ cells. β-galactosidase (β-gal) activity was also observed in most interstitial cells and seminiferous tubules at P3 (S1 Fig.), in accordance with Cyp19/aromatase expression in testes.

**Fig 2:**
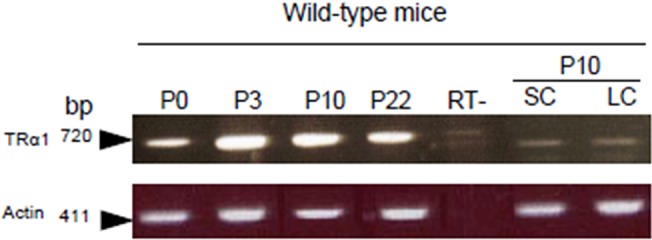
RT-PCR detection of TRα1 mRNA in testes during post-natal development in wild-type mice. TRα1 mRNA (720 pb) was present in whole testes from birth (P0) to P22, and in SC- and LC-enriched fractions prepared from testes of wild-type mice at P10. TRα1-specific primers were described previously [27] (Table 1). Actin mRNA (411 pb) was detected to check RNA quality. RT-, control without RT.

Next we demonstrated that the *TRα1* gene was expressed in mice by LC and SC during post-natal development (Fig. 2).

We then aimed to produce transgenic mice with impaired function of this receptor in both cell types to compare these mice with the previously established TRα^AMI^-SC [8]. After having checked that ARO-iCre was useful for Cre-loxP excision in both SC and LC cells (Fig. 1C), we produced TRα^AMI^-ARO double transgenic mice as described in materials and methods. Littermates with the unrecombined TRα^AMI^ allele and negative for ARO-iCre served as control mice. Testicular function was then studied at physiological, cellular and hormonal levels. A summary of the results is presented in Table 2.

**Table 2:** Physiological, endocrine and cellular characteristics in TRα^AMI^-ARO (present study), TRα^AMI^-SC and TRα^Null/Null^ transgenic lines.

Mutant strain	TRα^Null/Null^	TRα^AMI^-SC	TRα^AMI^-ARO
Mutation	Gene knockout [27]	Dominant negative knock-in [25]	Dominant negative knock-in [25]
References (phenotype)	[7, 8]	[8]	Present study
Target cells	ubiquitous	Sertoli	Sertoli & Leydig
Adult testis weight (% change)	+26.7% ***	+14.2% ***	+16.5% ***
Testicular sperm reserve	increased ***	increased **	increased ***
SC proliferation index (P3)	increased ***	increased ***	increased ***
Histology of seminiferous tubules	unchanged [7]	unchanged	unchanged
Blood testosterone level (basal and stimulated)	normal	normal	normal
Blood LH level	ND	ND	normal

**P < 0.01;

***P < 0.001;

ND, not determined.

### TRα^AMI^-ARO males present increased sperm reserve and normal fertility

**Fig 3:**
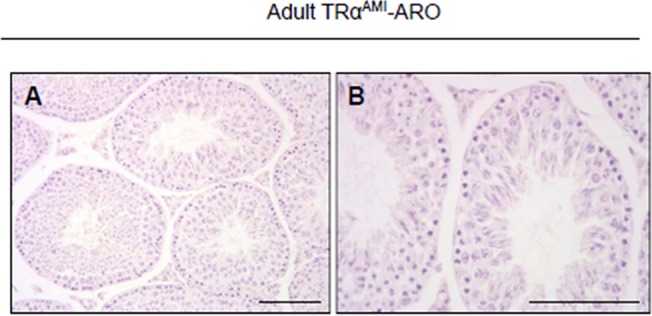
Histological sections of adult TRα^AMI^-ARO testes at low (a) and high (b) magnification. Seminiferous tubules were fully developed in TRα^AMI^-ARO. Their epithelium exhibited normal structure and organization. Hematoxylin staining. Bar represents 100 μm.

**Fig 4:**
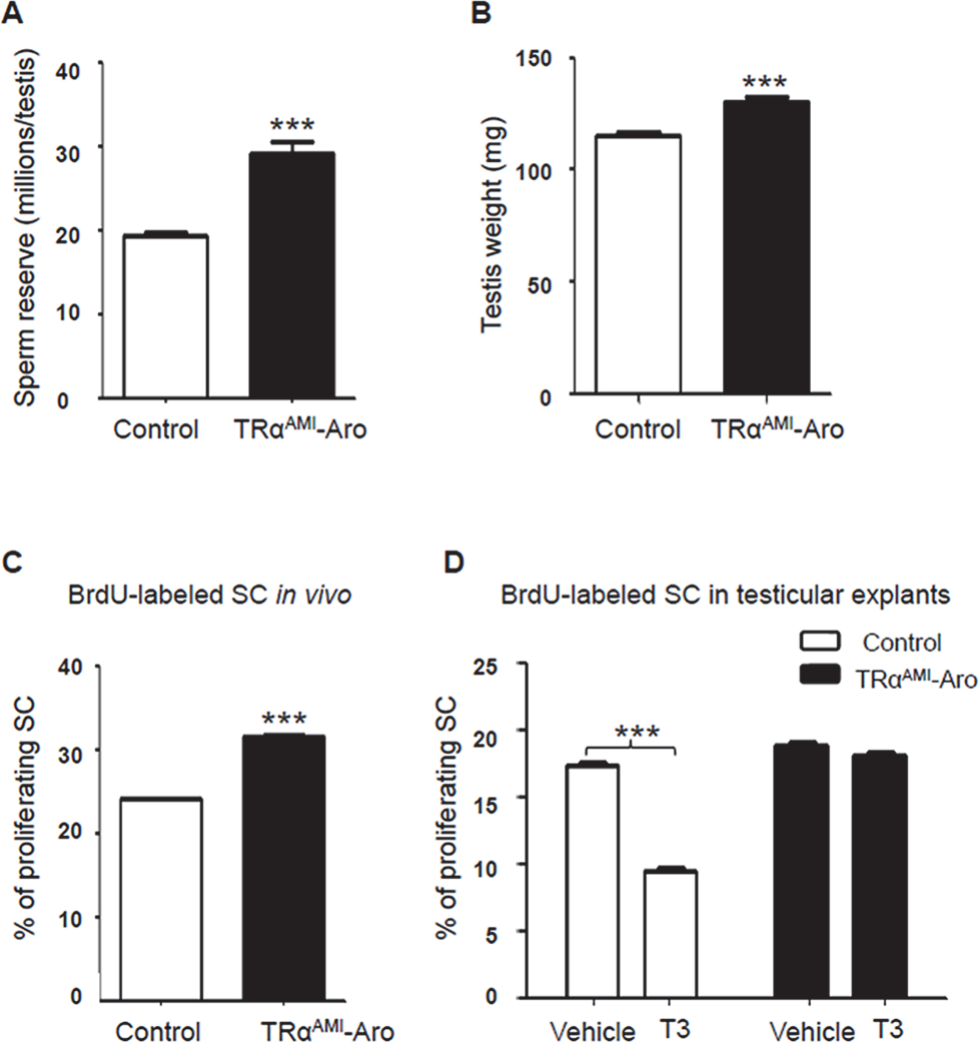
Whole testicular sperm reserve and testis weight in adult TRα^AMI^-ARO, and percentage of proliferating SC in TRα^AMI^-ARO at P3 *in vivo* and in testicular explants using organotypic *in vitro* cultures with or without exogenous T3. We observed a significant increase in whole testicular sperm reserve **(a)** and in testis weight **(b)** (***P < 0.001; n = 15 for controls and n = 16 for TRα^AMI^-ARO group). Data are shown as mean ± SEM. Statistical analyses were performed using Student’s *t*-test. White bars, control; black bars, TRα^AMI^-ARO (a, b). After BrdU immunohistochemical labeling, BrdU negative and BrdU positive SC were counted and the SC proliferation index was calculated. **(c)** When BrdU was injected *in vivo* 3 h before sacrifice, proliferation of Sertoli cells increased in P3 testes of TRα^AMI^-ARO mice in comparison with the control (***P < 0.001; n = 5 animals for each genotype). **(d)** When BrdU was added *in vitro* 3 h before the end of the organotypic cultures of P3 testicular explants, the SC proliferation index significantly decreased in the control in presence of T3 compared with vehicle (***P < 0.001, n = 6). In contrast, T3 had no effect on SC proliferative index in TRα^AMI^-ARO mice (*P* > 0.05, n = 5). Data are shown as mean ± SEM. Statistical analyses: two-way ANOVA followed by Bonferroni’s *post-hoc* test. White bars, control; black bars, TRα^AMI^-ARO.

We were particularly interested to find out whether the presence of the TRα^AMI^ allele in both SC and LC has the same consequences in adults as in TRα^AMI^-SC males, in which testis weight was high due to an increase in sperm reserve. Adult TRα^AMI^-ARO males were normally fertile (TRα^AMI^-ARO, 12.6 ± 1.2 pups per male; control, 12.0 ± 1.6 pups per male; n = 10 males per genotype). Histological analyses revealed no visible alteration of the seminiferous tubule epithelium (Fig. 3). In TRα^AMI^-ARO males we observed a significant increase (*P* < 0.001) in the total testicular sperm reserve (Fig. 4A) and in testis weight (Fig. 4B) in comparison with the control.

### SC proliferation index increased in TRα^AMI^-ARO just as TRα^AMI^-SC mice

In TRα^AMI^-SC males, we previously observed that the increase in sperm reserve resulted from an augmentation in the SC proliferation rate during post-natal development. The next step was to ask if the TRα^AMI^ allele in both SC and LC accentuate these cellular changes. To reach this aim, we investigated the percentage of SC incorporating BrdU within 3 hours at P3, *in vivo* and *in vitro*. *In vivo*, we found the SC proliferation index was significantly higher (*P* < 0.001) in TRα^AMI^-ARO testes than in control testes at P3 (Fig. 4C). We then wanted to know if the regulation exerted by TRα1 on the SC proliferation index described above was modulated in the presence of T3. To do so, we used organotypic cultures in which SC proliferation was measured in testes explanted at P3, in the presence or absence of exogenous T3. In organotypic cultures, SC proliferation without T3 (vehicle) was similar for TRα^AMI^-ARO and control testes, indicating that the TRα1 dominant-negative did not alter SC proliferation in the absence of T3 *in vitro* (Fig. 4D). As expected, addition of T3 significantly (*P* < 0.001) decreased (approximately two-fold) SC proliferation in the controls, whereas this response was fully abolished in TRα^AMI^-ARO.

### Testosterone and LH levels are unchanged in adult TRα^AMI^-ARO mice

**Fig 5:**
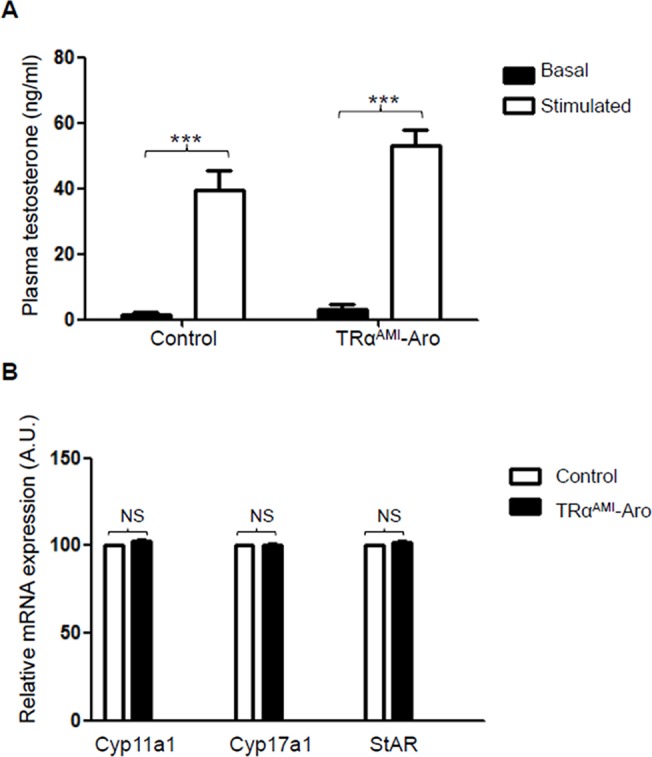
Plasma testosterone levels in adult TRα^AMI^-ARO males, in basal conditions and after hCG stimulation, compared with control mice and mRNA levels of Cyp11a1/P450ssc, Cyp17a1/P450c17 and StAR in TRα^AMI^-ARO testes. **(a)** In both TRα^AMI^-ARO (11 males) and controls (9 males), hCG induced a significant increase (***P < 0.001) in testosterone secretion, as expected. Plasma testosterone levels were unchanged in TRα^AMI^-ARO compared with the control. Data are shown as mean ± SEM. Two-way ANOVA followed by Bonferroni’s *post-hoc* test. Black bars, basal levels; white bars, hCG stimulated. **(b)** The amount of cDNA of 3 steroidogenic genes (Cyp11a1/P450ssc, Cyp17a1/P450c17 and StAR) was compared in TRα^AMI^-ARO (black bars) and control males (white bars). For each genotype, 6 mice were tested in triplicate using TaqMan quantitative real-time PCR, as described in materials and methods. Normalization was performed with two housekeeping genes, β-actin and GAPDH, that both exhibited similar expression among samples. mRNA expression is presented as percent of mean control level. A non-parametric test (Mann-Whitney) was used for statistical analysis. NS: not significant.

We then tried to find out whether the TRα^AMI^ allele present in LC affects their steroidogenic activity, compared with the TRα^AMI^-SC phenotype, where plasma testosterone levels were unchanged. For this, we measured basal and hCG-stimulated testosterone levels. Both basal and hCG-stimulated testosterone levels were similar in adult TRα^AMI^-ARO and the controls, with significant effects of hCG stimulation in both lines, as could be expected (Fig. 5A).

To consolidate the androgenic status of TRα^AMI^-ARO, we investigated mRNA levels of steroidogenesis enzymes P450c17 (Cyp17a1), P450scc (Cyp11a1) and StAR per testis, which were all unaffected (Fig. 5B). Moreover, as expected, LH levels were similar in TRα^AMI^-ARO males and control males (0.85 ng/ml ± 0.4 in TRα^AMI^-ARO *versus* 0.63 ng/ml ± 0.2 in control, n = 6 for each genotype).

## Discussion

### A new genetic mouse model to study testicular somatic cells

We previously used the TRα^AMI^ allele that induces constitutive T3-regulated gene repression by preventing interaction with coactivators [25,32]. We used the TRα^AMI^ allele to demonstrate that T3 controls post-natal SC proliferation through TRα1 in a direct and cell-autonomous manner during early post-natal development [8]. Here, we present the introduction of this conditional dominant-negative allele in post-natal testes in both LC and SC compartments using the novel ARO-iCre transgenic mouse strain generated in our lab.

We showed that Cre activity of the ARO-iCre line was restricted to male and female gonads, confirming the highly specific Cyp19/aromatase(IIa)_-278_ promoter expression profile [23]. In contrast, other available gonad-specific Cre transgenic lines driving recombinase expression in steroidogeneic cells, are also active in cells from others tissues, namely adrenal tissue and hindbrain for the Cyp11a-GC (GFP-Cre) line [33], and male adrenal tissue for the Cyp17-iCre line [34]. In the literature, the anti-Müllerian hormone type 2 receptor (AMHR2)-Cre line [35] was mostly used to target conditional gene mutation in LC, for instance for the genes coding for SF1 [36], androgen receptors [37] or ALK3 [38], but also in SC, for example to impact WNT/beta-catenin signaling [39].

We characterized testicular iCre activity using a ROSA26 Cre reporter mouse. We detected loxP recombination in both SC and LC at P3, an age when SC are highly proliferative. This result is fully coherent with the aromatase expression profile observed in post-natal testes. Aromatase activity corresponds to the irreversible conversion of androgens into estrogens [40]. In LC, this activity increases progressively from birth to puberty as the testis matures [41,42]. In SC, which only express aromatase during post-natal development, activity decreased 2-fold from birth to 2 weeks of age. Consequently, estrogens are almost exclusively produced by LC during adulthood [43].

In our model, we observed a phenotype similar to TRα^AMI^-SC obtained with AMH-Cre which is known to target all SC, suggesting that the ARO-iCre is rapidly active in the majority of post-natal SC. The absence of ARO-iCre activity in male germ cells, evidenced by a lack of β-galactosidase staining and no transmission of the deleted allele to offspring (data not shown), is in full agreement with Hinshelwood et al. [23], who showed that the Cyp19/aromatase(IIa)_-278_ promoter did not target Cre in oocytes.

### Phenotype of TRα^AMI^-ARO males and parallel with TRα^AMI^-SC

We compared the TRα^AMI^-ARO line, which presents a constitutive repression of the T3/TRα1 pathway in LC and SC—a kind of local hypothyroidism—with the TRα^AMI^-SC and TRα^Null/Null^ lines (summarized in Table 2). In early post-natal TRα^AMI^-ARO testes, the SC proliferation rate was similar to the rate observed in TRα^AMI^-SC of the same age, which explains why the increase in sperm reserve and in testis weight are of similar magnitude in adulthood in both lines. These results are not surprising since the KO of *THRA* (TRα^Null/Null^ line) led to similar effects.

In adult TRα^AMI^-ARO males compared with their controls, we did not observe any change in plasma testosterone and LH levels, nor in steroidogenesis enzyme expression in testes. These converging observations support that local hypothyroidism induced before puberty by a dominant-negative TRα1 receptor does not influence LC steroidogenic activity, directly or indirectly, through putative SC-pathways. In the literature, on experiments in rats and humans, and despite abundant documentation of T3-dependent regulation of LC precursor cell differentiation [44,45], this point was controversial, depending on the model and the experimental approach used. In rats made hypothyroid with propil-thiouracil treatment during post-natal development or adulthood, it was observed that peripheral testosterone concentrations were unchanged [4,16,17,46,47]. In contrast, more recent studies on neonatal rats made hypothyroid using methimazole showed a different result, concluding that T3 increases testosterone production [18,19]. Yet another study, using thyroidectomy and thyroid hormone supplementation, showed a negative influence of T3 on testosterone production, along with decreased LH and FSH levels [48]. The degree of hypothyroidism could influence the impact on steroidogenesis and discrepancies may be due to the various molecules used to develop hypothyroidism, and/or by the timing of the pharmacological treatment. In humans as well, the impact of T3 deregulation varies from case to case (for review [49]), with decreased [50,51] or unchanged [52] blood testosterone levels in hypothyroid men. Our results suggest that the testosterone and LH endocrine disturbances observed in various hypothyroid animals (including humans) are probably more related to a disturbance at central level of the hypothalamo-pituitary axis than at gonadal level. Moreover, the discrepancies between pharmacological and functional models suggest that such perturbations may be the result of cumulative effects of T3 *via* TRα1 and possibly TRβ1, as we discussed in our previous work [8]. Indeed, whereas TRβ2 appears to be restricted to the nervous system, TRβ1 is expressed in various testis cells. Therefore, this receptor may directly transduce certain T3 functions in LC and/or SC, in addition to TRα1 (for review see [53,54]). It is possible that TRβ1 may palliate TRα1 in case of loss of receptor function. As discussed in our previous work, the systematic analysis of mice expressing the TRα^AMI^ allele in various tissues suggests that most TRβ functions are preserved in this model [25,55], explaining the fact that the TRα^Null/Null^ testis phenotype does not fully recapitulate post-natal hypothyroidism. In particular, in the present TRα^AMI^-ARO, TRβ1 could have significant function in Leydig cells, equilibrating testosterone levels.

A final point is the role of SC paracrine factors in the regulation of LC, with some of them putatively regulated by T3 during post-natal development, such as insulin-like growth factor-1 [54,56]. However, the TRα^AMI^-ARO phenotype suggests that such regulation through TRα1 is negligible, and this result is concordant with our previous study in which TRα^AMI^ in SC alone did not affect testosterone levels.

## Conclusion

Our work has confirmed the role of TRα1 in SC during post-natal life and pointed out that T3 does not, or only moderately, regulate steroidogenic activity through this receptor, whether in a direct LC-autonomous manner, or indirectly through SC paracrine regulation. This study also brings forward an additional genetic tool (i.e. the ARO-iCre line) to investigate LC function. Further characterizations of this model should define precisely the timing of Cre activity and its localization in the ovary.

## Supporting Information

S1 Fig characterization of ARO-iCre transgenic mouse line at P3. Cre recombinase activity detected in somatic cells of testes in ARO-iCre males at P3. After crossing with a ROSA26 Cre reporter mouse, β-galactosidase activity was detected in both ST (seminiferous tubules) and In (interstitium). Bar represents 50 μm.
(TIF)
