# Supplementary figures and images for: Expression of Dominant-Negative Thyroid Hormone Receptor Alpha1 in Leydig and Sertoli Cells Demonstrates No Additional Defect Compared with Expression in Sertoli Cells Only
Source: PLoS One. 2015 Mar 20;10(3):e0119392. doi: 10.1371/journal.pone.0119392 (PMC4368620; doi:10.1371/journal.pone.0119392)

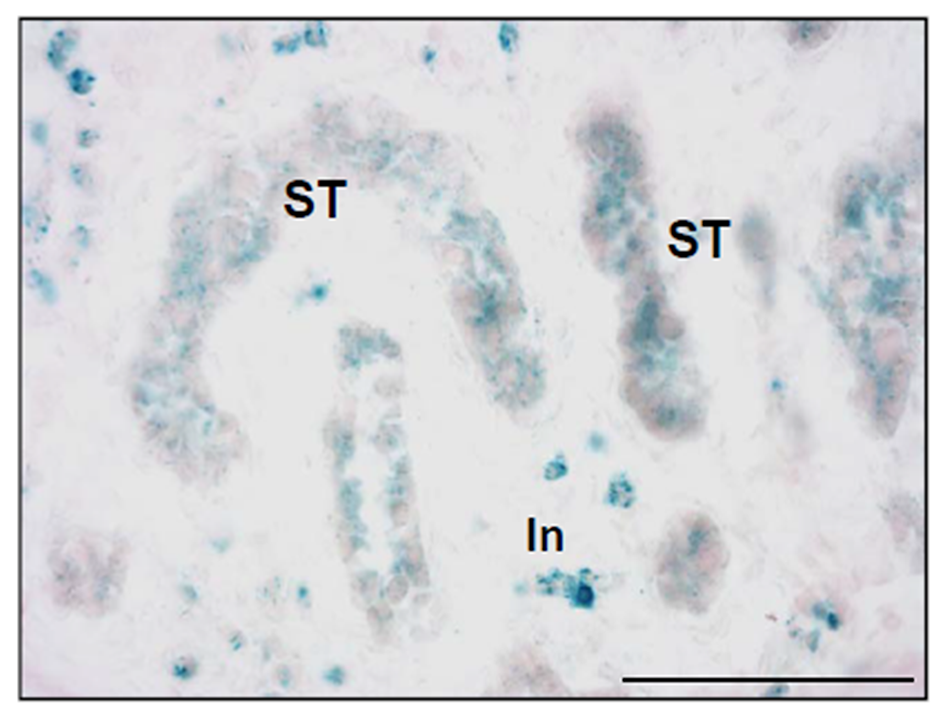

Supplement: S1 Fig — Cre recombinase activity detected in somatic cells of testes in ARO-iCre males at P3. After crossing with a ROSA26 Cre reporter mouse, β-galactosidase activity was detected in both ST (seminiferous tubules) and In (interstitium). Bar represents 50 μm. (TIF) [file pone.0119392.s001.tif]
